# Brain-derived signals related to ball kicking movement in soccer and technologies employed: a systematic literature review with gap map

**DOI:** 10.1186/s13102-026-01676-y

**Published:** 2026-04-01

**Authors:** Nicole Unsihuay, Filipe Manuel Clemente, Robert Trybulski, Rohit Kumar Thapa, Murilo Henrique Faria, Fanny Lys Casado, Fernando Zvietcovich, Luiz H. Palucci Vieira

**Affiliations:** 1 Departamento Académico de Ingeniería, Sección de Bioingeniería, Research Group on Technology Applied to Health and Physical Performance – TeHealP@PUCP, Pontificia Universidad Católica del Perú, Lima, Peru; 2 Department of Biomechanics and Sport Engineering, Gdansk University of Physical Education and Sport, Gdansk, Poland; 3 Sport Physical Activity and Health Research & Innovation Center, Coimbra, 3045-601 Portugal; 4 Applied Research Institute (i2A), Polytechnic University of Coimbra, Coimbra, 3045-601 Portugal; 5 Medical Department, Wojciech Korfanty Upper Silesian Academy in Katowice, Katowice, 40-659 Poland; 6 Provita Żory Medical Center, Żory, Poland; 7 Symbiosis School of Sports Sciences, Symbiosis International (Deemed University), Pune, India; 8 São Paulo State University (UNESP), Campus Bauru, Bauru, 17033-360 Brazil

**Keywords:** Measurement, Portable neuroimaging systems, EEG, fNIRS, Team sports, Biomechanics

## Abstract

**Background:**

Recent technological advances have enabled the development of portable data acquisition systems that facilitate the collection of brain signals during sports tasks. The main objective of the present systematic review was to collate evidence regarding studies that have analysed brain-derived indices related to ball kicking action.

**Methods:**

The PRISMA guidelines were followed in the protocol for this review. Six electronic databases were searched (IEEE Xplore, Scopus, Web of Science, APA PsycNet, EBSCOHost, and PubMed). The search string followed a PICOS/PECOS framework: participants as human able-bodied subjects regardless of age, evaluated while performing a ball kick task, and reported results of brain-derived metrics. The RoBANS tool was used to evaluate the risk-of-bias of the included studies.

**Results:**

The database searches resulted in a total of 1748 records, of which 8 original research articles met all the inclusion criteria. Most studies used EEG systems while few employed fNIRS. Qualitative synthesis indicated that skilled ball kicking performance was generally accompanied by phase-specific cortical dynamics (e.g., within frontal, sensorimotor/central, and parieto-occipital regions) whereas anxiety and injury appear to shift cortical engagement toward potentially compensatory, less efficient control strategies. Data on measurement error was limited while blinding aspects were frequently omitted across studies. Finally, one problem identified in this review was that only one-fourth studies used an opponent attempting to block the shots.

**Conclusions:**

Preliminary evidence suggest that kicking outcomes tended to be linked with specific brain patterns. Future studies need to improve the design of experimental tasks so that they more closely resemble what occurs in a real game.

**Trial registration:**

The review protocol was registered in OSF Preregistration under ID #NZASB.

**Supplementary Information:**

The online version contains supplementary material available at 10.1186/s13102-026-01676-y.

## Introduction

Ball kicking has been a determinant variable of match performance (i.e. outcomes) in male and female youth or senior soccer, given the strong influence of shooting on target upon winning results [1–6], motivating extensive scientific analysis of the movement [7]. Indeed, the kicking action consists in the most widely studied skill across the biomechanics of soccer literature, reflecting its technical importance and recurrent use across playing situations [8]. Ball kicking is characterized as a multiarticular ballistic action, involving proximal-to-distal motion sequencing of the lower-limb segments that may directly influences on the development of foot velocity and ball placement-derived outcomes [9, 10]. Throughout the history of biomechanical - and motor control - analysis of kicking in soccer, the methodological progress from 2D (two-dimensional) kinematics toward 3D (three-dimensional) full-body biomechanics has improved descriptive and explanatory models of kicking, while simultaneously emphasizing the need to capture movement coordination across the whole body and task phases [11]. Deterministic models [12] have indeed been previously tested regarding the mechanical factors related to ball kicking outcomes in both youth and senior players [13, 14]. Nevertheless, even though there is a mature knowledge base on the characterization of biomechanical features and the effects of individual and environmental constraints upon soccer ball kicking [7, 9, 11, 15, 16], understanding of brain–behavior relationships in ecologically valid sports actions remains comparatively limited, particularly for high-speed skills executed under accuracy and pressure constraints [17].

Skilled motor performance depends on distributed central cortical networks that support voluntary movement control and exhibit experience-dependent plasticity [18]. In general, motor skill acquisition occurs through time-dependent stages that include rapid within-session gains and later consolidation processes that stabilize performance [19]. Furthermore, accurate skilled actions further rely on adaptive internal models that use sensory prediction errors to attempt refine motor commands and maintain calibration [20]. In electrophysiological terms, self-initiated actions are preceded by the Bereitschaftspotential (i.e. early cortical activation preceding self-initiated movements), reflecting preparatory activity across medial and lateral motor areas with temporospatial structure related to movement generation [21]. Complementarily, event-related desynchronization/synchronization in mu (standard alpha band) and beta rhythms provides time-resolved indices of sensorimotor activation and inhibition during preparation, execution, and imagery [22]. Lower-limb movements also exhibit somatotopically organized oscillatory dynamics over sensorimotor regions, underscoring the feasibility of tracking leg-related cortical processes using scalp electroencephalogram (EEG) [23]. 

Since many neuroimaging paradigms constrain natural movement, mobile brain/body imaging concepts have been proposed to link distributed brain dynamics to real-world action while recording behavior with high bandwidth [24]. Mobile brain imaging specifically enables simultaneous recording of EEG with body dynamics during active behavior, and this may support study designs with improved ecological validity [25]. Advances in lightweight systems and synchronized multimodal acquisition have further operationalized “natural cognition in action” approaches that integrate EEG with motion and physiological measures [25]. However, movement-induced artifacts and other non-neural contamination typically increase with movement intensity, and methodological consensus on optimal mitigation strategies remains incomplete [26]. Foundational demonstrations show that artifact attenuation (e.g., template regression coupled with independent component analysis (ICA)) can recover meaningful EEG features during whole-body locomotion, supporting the broader feasibility of mobile EEG in dynamic tasks [27]. At the same time, semi-periodic movement artifacts can sometimes produce components that resemble plausible brain sources, requiring careful validation when interpreting source-resolved results [28].

As concerning soccer-specific kicking studies, recent portable EEG experiments have begun to evaluate/explore the potential link between brain-derived signals and performance outcomes [29–31]. Despite this growth, mobile EEG in movement contexts still lacks broad consensus for quantifying and characterizing artifact burden [32]. In parallel movement domains, systematic evidence on EEG–EMG connectivity shows substantial methodological variability and explicitly calls for better alignment of approaches, reinforcing the likelihood of similar harmonization needs in kicking research [33]. Alongside EEG, portable hemodynamic imaging such as near-infrared spectroscopy has long been positioned as a practical approach for studying cortical involvement during exercise and motor tasks in moving humans [34]. In movement science, functional near-infrared spectroscopy (fNIRS) applications have also been expanded, despite with heterogeneous protocols and data-processing choices which may limit comparability and interpretability across studies [35]. Consensus-oriented recommendations for fNIRS in posture and gait explicitly emphasize standardized conduct, artifact handling, and reporting, and such issues have been directly relevant when translating neuroimaging to complex sport skills [36]. These developments indicate a timely need to consolidate what is known about brain-derived signals during soccer kicking and the technologies used to measure them, to better support performance science and rehabilitation translation [37]. With these assumptions in mind, the current literature review aims to (i) systematically collate and critically evaluate the evidence on brain-derived signals reported in original research studies in relation to ball-kicking action in soccer and (ii) map the acquisition technologies and analytical pipelines used to capture and process these signals. We additionally aim to identify current methodological and translational gaps and derive evidence-informed directions for future neuro-mechanical research on soccer kicking.

## Materials and methods

The protocol for the present review followed the items [Additional file 1] suggested by The Preferred Reporting Items for Systematic reviews and Meta-Analyses (PRISMA) statement [38] and was registered prior to the execution of the methodology processes (2025-06-09 04:03 PM GMT − 5) in the OSF – Open Science Framework under ID #nzasb (DOI:10.17605/OSF.IO/NZASB), considering the structure according to a recently published systematic review [39]. All the papers included in the current systematic review (i.e., qualitative synthesis of evidence) reported ethical aspects adopted for data collection in its full-texts and this was defined in as an inclusion criterion in the aforementioned protocol.

### Search strategy

The electronic databases IEEE Xplore, Scopus, Web of Science, APA PsycNet^®^, EBSCOHost, and PubMed were searched on 12/06/2025 (between 03:21 to 07:56 PM GMT − 5) in an attempt to identify evidence about brain-derived signals related to ball kicking movement in soccer, published as articles within scientific journals. The search strategy and key terms were defined according to previous systematic reviews that analyzed on a separate basis the brain activity or ball kicking in the soccer context [15, 40, 41]. Using a Boolean search strategy, the final search string consisted of (soccer OR football* OR association football OR 11-a-side) AND (kick* OR shoot* OR pass* OR ball handling OR ball-kicking OR goal-directed OR skill OR technical) AND (brain OR cortex OR cortical OR neural OR neuronal OR EEG* OR electroencephalography OR fNIRS OR nirs OR functional near-infra*). The searches focused on the fields of title, abstract and keywords across all the aforementioned databases.

### Eligibility criteria

This review only considered original studies that were (i) scientific articles peer-reviewed; (ii) with abstract available for screening in the respective electronic database; (iii) full-text published in English language; using a PICOS/PECOS framework [42]: (iv) Participants – when included human able-bodied subjects (e.g. soccer players) regardless of age; (v) Intervention/Exposure – ball kick task; (vi) Comparator – not applicable; (vii) Outcomes – reported results of brain derived metrics (e.g. EEG power) and (viii) Study design – no restrictions were imposed. No restrictions were also outlined in reference to the date of publication (i.e., articles were searched from inception up to the date of search completion reported here).

### Study selection process

Initially, the search results from all the databases were imported into an open-source reference management software (Zotero v.7.0.15; Corporation for Digital Scholarship, USA), which was used for the execution of this whole protocol. Thereafter the duplicates were removed automatically using its “Duplicate Items” function. Following on two authors (N.V. and F.Z.) independently evaluated the title, abstract and keywords of all registries identified in the electronic database search. Discrepancies were resolved in an additional consensus round with a third author, that consists also in a senior researcher in the area (L.V.). The evaluators were instructed to consider the eligibility criteria mentioned above and also to not consider inclusion of studies (i) published as book chapters, conference proceedings, or similar types (i.e. grey literature); (ii) if the article was later retracted; (iii) experiments conducted without reporting of any ethical aspects in the article text or the eventual exoneration – if applicable [43, 44]; (iv) considered task movements pertaining to football codes distinct to soccer; (v) with no information concerning the method/equipment adopted to record brain activity or (vi) with no information about which specific brain regions were considered for data collection.

### Data extraction and evidence synthesis

The following information was extracted from included studies: publication details – authors, year of publication and funding; demographic characteristics – geographical location of the study, number of participants, sex, age, playing level and positional role; task protocol – location, instruction/aim, ball characteristics, limb(s) used; data outcomes – cortical areas evaluated, data collection device and acquisition frequency; signal processing techniques and pertinent findings. The threshold for judgement of a given study result as statistically significant was set at *p* ≤ 0.05 unless otherwise stated. These items were selected taking into account previous systematic reviews [15, 41, 45]. One author conducted the data extraction (M.F.) and a second author verified the extracted data (N.V.).

The STROBE checklist [46] and the RoBANS tool (Risk of Bias Assessment Tool for Nonrandomized Studies) [47] were used respectively to critically appraise the reporting completeness and risk-of-bias in results/inferences. In particular, the Items 1, 3, 6, 8, 12, 14, 18, 19, 20 and 22 derived from STROBE were selected for the present review. Each item was rated using a numerical scale (“completed” = 1 or “incomplete” = 0). Two authors independently evaluated each study individually (N.V. and F.L.C.) and a third author (L.V.) checked the ratings and resolved any discrepancies if existed. A sum of all items was then computed for each study. Based on that, the included studies were deemed to have high (∑ ≥ 8) or low (∑ ≤ 7) reporting completeness [48, 49]. In addition, all six original items from RoBANS were considered for assessments including selection of participants, confounding variables, measurement of exposure, blinding of outcome assessments, incomplete outcome data, and selective outcome reporting. Each item for each individual study was rated separately (by two authors: N.V. and M.F.) as presenting a low, high, or unclear risk of bias. If any discrepancy existed, a third senior author in the area (L.V.) was then consulted and resolved. The robvis tool was used to generate the corresponding plot of risk-of-bias outcomes [50].

Finally, gap maps were also constructed to assist provide a synthesis of the findings across studies as well as potential directives for future investigations [51]. The gap maps were organized as 2-dimensional matrices (rows = study outcomes or methodological characteristics; columns = contextual variables). Each cell in the respective matrix represents the number of studies - if any - found addressing such combination between the two given variables. Cell shading was used to indicate evidence density [– = no studies (empty cell); white plus number = 1–2 studies; dark gray plus number = ≥ 3 studies]. In particular, two main gap map categories were defined, including: (1) a matrix linking brain-derived outcomes investigated × brain regions explored in the included studies and (2) a matrix linking (a) - neuroimaging technologies × brain regions; (b) - kicking task × population; and (c) - key study design features x neuroimaging modalities used across the included studies.

## Results

**Fig. 1:**
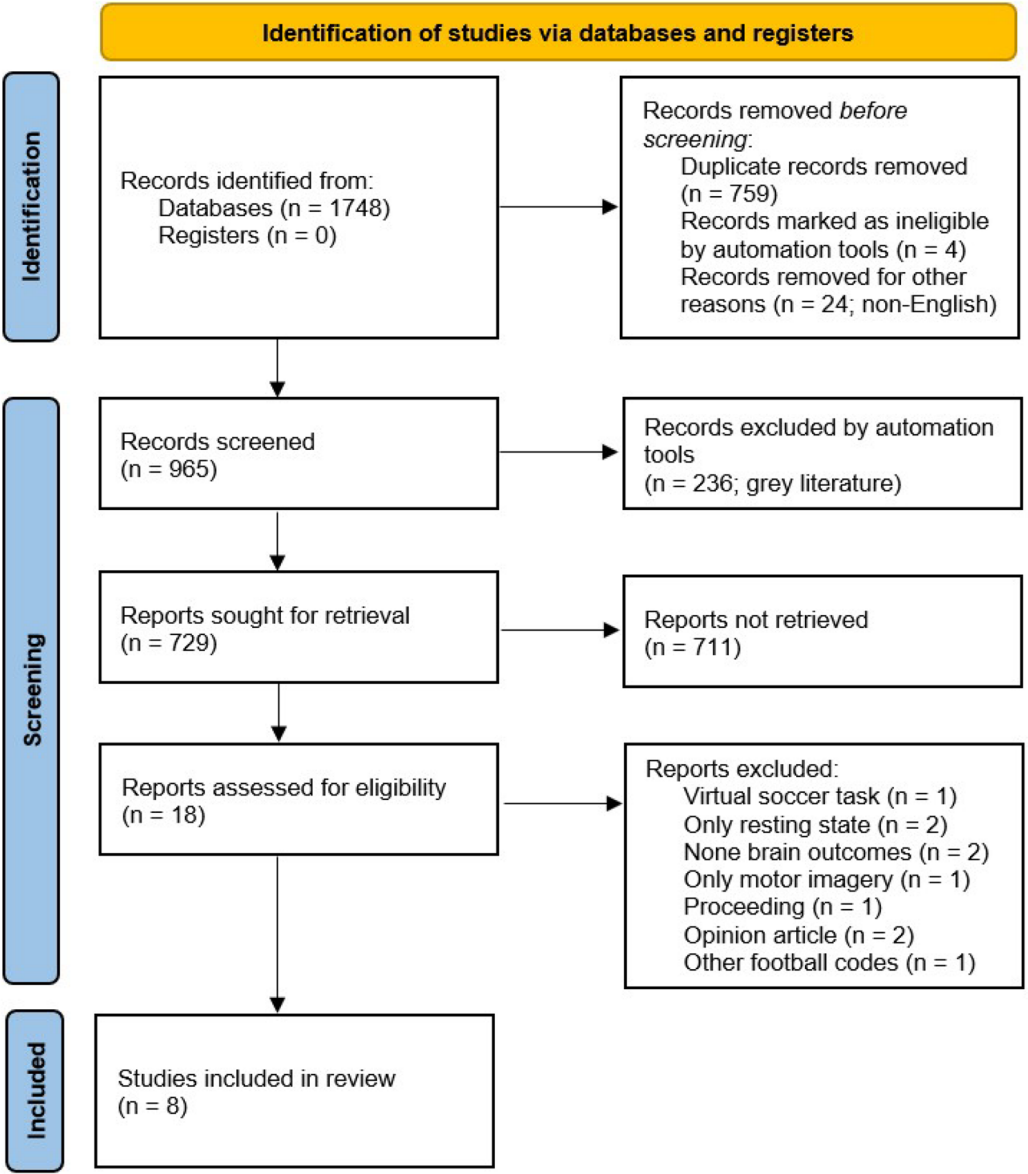
PRISMA 2020 flow diagram for the current systematic review on brain activity and ball kicking

A total of 1748 reference entries were initially identified when pooling all results of the searches in the databases (IEEE Xplore – *n* = 148, Scopus – *n* = 573, Web of Science – *n* = 451, APA PsycNet^®^ – *n* = 1, EBSCOHost – *n* = 408, and PubMed – *n* = 167). Next, 783 records were removed before screening due to automatic identification by the reference manager software as being duplicates (*n* = 759), published in a non-English language (*n* = 24) or retracted works (*n* = 4). Regarding the duplicates, the items deemed as the same indeed were merged and this process was supervised case-by-case. In the next stage, 236 reference entries were deleted owing to the fact that they were classified as grey literature, resulting in 729 records sought for retrieval. Following inspection of the titles, abstracts and keywords, 18 reports were assessed for eligibility, i.e. the step of full text analysis. After reading their full-texts, 8 [29–31, 52–56] were finally included in the review (Fig. 1). In the last screening stage, exclusions were due to (i) the use of a only virtual soccer task, (ii) only resting state measures reported, (iii) no brain outcomes reported, (iv) the use of only motor imagery paradigm, (v) paper published as conference proceeding, (vi) opinion piece article and (vii) covering other football codes.

### Main sample characteristics and purposes of studies

Table 1 presents the sample characteristics and purpose of the included studies. Of the eight studies (with 183 participants), six included seniors, and one included youth participants, while one study [52] did not provide information on the age of the participants. Four studies (50%) involved only male participants [29, 30, 52, 55], three (38%) included both male and female participants [31, 53, 54], and in one study this information was not reported [56]. All studies included semi-professional or non-expert players; none included professional or elite players. Two studies reported the inclusion of samples from all playing positions [29, 30], while in the remaining studies (75%), the participants’ positional roles were uncertain or unspecified. Sample sizes varied from 10 participants [30] to 39 participants [55]. The association between brain-derived signals and kicking outcomes was the most frequently addressed topic in the included studies (i.e., 5 out of 8 studies; four EEG [29–31, 52] and one fNIRS [56]). One of the early studies, conducted by Collins et al. [52] in 1991, investigated changes in cerebral activity (i.e., alpha frequency) during a ball-kicking task among non-soccer-playing, physically active male participants. The participants were instructed to kick a soccer ball from a distance of 7 m through a 30-centimeter-wide channel marked by two cones, while their cerebral activity was being recorded.

**Table 1:** Sample characteristics and purposes of the included studies

Reference	Year	Location	Study purpose	*N*	Gender	Age	Playing level	Playing position(s)
Collins et al. [52]	1991	UK	To investigate whether the changes in the alpha frequency band observed in experts during the karate modality also occurred in individuals relatively novice to the kicking task.	22	Male	--	Sub-elite	unspecified
Li et al. [30]	2025	Germany	To investigate whether the changes in the 8–13 Hz frequency band are involved in successful and unsuccessful penalty kicks in skilled soccer players.	10	Male	Senior	Sub-elite	All (pooled)
Palucci Vieira et al. [29]	2022	Brazil	Identify the magnitude of possible associations between the electroencephalographic signals and lower limb kinematic parameters during ball kicking action	24	Male	Youth	Sub-elite	All (pooled)
Piskin et al. [53]	2024	Germany	To investigate the cortical dynamics involved in target-directed kicking based on source-based analysis.	11	3 female/8 male	Senior	Sub-elite	unspecified
Piskin et al. [54]	2025	Germany	To compare the kicking performance and associated cortical activity between injured and healthy soccer players.	25	10 female/ 15 male	Senior	Sub-elite	unspecified
Piskin et al. [31]	2024	Germany	To compare the neural mechanisms involved in passing between novice and experienced players.	30	13 female/17 male	Senior	Sub-elite	Unspecified
Schmaderer et al. [55]	2023	Germany	To investigate the prefrontal activity of soccer experts during general and sport-specific cognitive tasks.	39	Male	Senior	Sub-elite	Unspecified
Slutter et al. [56]	2021	Netherlands	To compare brain activity during a penalty kick in which soccer players felt anxious and not anxious, using fNIRS.	22	--	Senior	Sub-elite	No

Palucci Vieira et al. [29] in 2024 studied the association between brain oscillations at different frequency bands (i.e., delta, theta, alpha, beta, gamma) across different regions of the brain (i.e., frontal, motor, parietal, and occipital) during three phases of soccer kicking (i.e., preparation phase, approach phase, and immediately before ball contact phase) and ball velocity and mean radial error. The authors [29] involved 24 male U17 soccer players competing in regional-level competitions. In another study, Li et al. [30] in 2025 investigated EEG power in the 8–13 Hz frequency band at frontal and central regions before penalty kicks, comparing successful and unsuccessful attempts. The study [28] involved 10 right-footed male skilled soccer players who participated in regional-level tournaments in Germany.

Piskin et al. [53] in 2024 investigated cortical dynamics (measured using EEG) during soccer kicking, specifically aiming to pass the ball towards a target located three meters away. In another two studies, one also in 2024 and another in 2025, the authors used similar methodological procedures [27] and compared the cortical dynamics during soccer kicking between experienced and novice participants (Piskin et al. [31]) as well as between participants with an injury (i.e., anterior cruciate ligament-reconstructed players) and healthy individuals (Piskin et al. [54]).

Schmaderer et al. [55] in 2023 investigated the cortical processing of experienced soccer players during general and sport-specific tasks. The participants were involved in a sport-specific task requiring them to pass the ball against one of the three back-pass walls. A green square was the signal to pass amongst the three target walls. For the general cognition test, the participants were asked to stand in front of a 2 × 3 m wall and choose the accurate visual stimuli. A total of eight visual stimuli generators were present, and one color would change, and the participants had to move their hands in front of the stimulus. Slutter et al. [56] in 2021 compared brain activity during penalty kicks with a friendly goalkeeper, an amiable goalkeeper, and a competitive goalkeeper. In addition, the authors [56] compared the brain activity during these penalty kick conditions between experienced and inexperienced participants. The authors [56] also investigated the association between anxiety and brain activity during these penalty kick conditions.

### Overview of data collection methods and technologies used

Table 2 presents the data collection procedures and technologies used in the included studies. In this sense, two neuroimaging modalities were identified across studies: electroencephalography (EEG) and functional near-infrared spectroscopy (fNIRS). The earliest study by Collins et al. [52] did not specify the brand of the EEG used to collect the data. Three studies [31, 53, 54] used EEG system from antiCap (Brain Products, Germany) while two studies used ANT Neuro (ANT Neuro b.v., Netherlands; Germany) [29, 30]. Regarding fNIRS, one study each utilized NIRx technologies (NIRx Medical Technologie, USA) [55] and Artinis Brite (Artinis Medical Systems, The Netherlands) [56]. The acquisition frequencies for EEG studies ranged from 250 Hz [52] to 1024 Hz [29] and most studies (4 out of 8 studies) used 500 Hz. The acquisition frequencies for fNIRS studies ranged from 8.7 Hz [55] to 10 Hz [56]. The experimental protocol used in the included studies varied, with only three studies [31, 53, 54] using similar protocols. Regarding the number of trials, these ranged from 6 trials [52] to 90 trials [31, 53, 54], with most studies using between 15 and 40 trials [29, 30, 55, 56]. Most studies evaluated the preferred limb, and only one study evaluated both the preferred and non-preferred limbs [52], while another study did not specify this methodological aspect [55]. The testing was conducted in laboratory conditions in three studies [31, 54, 55], and in outdoor conditions in two studies (artificial soccer pitch [56], and on natural grass pitch [29]), while three studies did not specify the environmental conditions [30, 52, 53].

**Table 2:** Data collection procedures and technologies used in the included studies

Reference	Technology system used to collect brain data	Acquisition frequency	Brain regions of interest (ROIs)					Experimental protocol	Limb evaluated	Environment	Opponent presence	Technology used for additional measures
			Frontal	Central	Parietal	Temporal	Occipital					
Collins et al. [52]	EEG - unspecified	250 Hz		✓	✓	✓		6 trials (three on each foot) kicking a stationary soccer ball between two cones placed 30 cm apart at a distance of 7 m	Both preferred and non-preferred	Unspecified	No	--
Li et al. [30]	EEG - eego (ANT Neuro, Germany)	500 Hz	✓	✓				30 trials (Divided into 3 blocks of 10 kicks, interspersed with 10 min of rest). Aiming to kick at a goal from a distance of 1,100 cm. The target size was adjusted to achieve a kicking success rate between 40 and 60%	Preferred	Unspecified	No	--
Palucci Vieira et al. [29]	EEG - eegoTM sports (LE-200, ANT Neuro b.v., Enschede, Netherlands)	1024 Hz	✓	✓	✓		✓	20 trials of 18-m instep kicks in a stationary ball aiming at 1 × 1 m targets positioned in the goalpost upper corners	Preferred	Natural grass soccer pitch	Yes - GK	3D kinematics (4 GoPro^®^ Hero 7 cameras, GoPro GmbH, München, Germany − 240 Hz) for tracking lower limb markers and 2D kinematics (2 GoPro^®^ cameras − 60 Hz) for tracking ball centroid
Piskin et al. [53]	EEG (antiCap, Brain Products, Germany)	500 Hz	✓		✓		✓	90 trials were made in 6 blocks of 15 trials. The target (a wooden box fixed to the ground (10 × 9 × 15 cm)) was positioned three meters away	Preferred	Unspecified	No	7 Wearable IMUs (myoMOTION, Noraxon, USA) were used on the kicking leg and supporting leg: pelvis, thighs, shanks, and feet. Webcam (Logitech Brio, Switzerland) for video recording, precision performance, synchronized with myoRESEARCH software
Piskin et al. [54]	EEG (antiCap, Brain Products, Germany)	500 Hz	✓		✓		✓	90 trials were made in 6 blocks of 15 trials. The target (a wooden box fixed to the ground (10 × 9 × 15 cm)) was positioned three meters away	Preferred	Laboratory	No	7 Wearable IMUs (myoMOTION system, Noraxon, USA) were used bilaterally, attached to the feet, shanks, upper thighs, and sacral surface of the lumbar area
Piskin et al. [31]	EEG (antiCap, Brain Products, Germany)	500 Hz	✓		✓		✓	90 trials were made in 6 blocks of 15 trials. The target (a wooden box fixed to the ground (10 × 9 × 15 cm)) was positioned three meters away	Preferred	Laboratory	No	Wearable IMU system (myoMOTION, Noraxon, USA); 6 sensors were used bilaterally attached on the feet, shanks and upper thighs, whereas the pelvis sensor was placed on the sacral surface of the pelvis
Schmaderer et al. [55]	fNIRS (NIRSport, NIRx Medical Technologies LLC, New York, USA)	8.7193 Hz	✓					40 passes. The participant must pass the ball as quickly as possible against one of the three back walls, positioned in a semicircle at 0°, 45°, and 90° angles around the participant, with a radius of 4 m	--	Laboratoty	No	Vienna Test System (Schuhfried GmbH, Mödling, Austria) to measure reactivity and decision-making; Witty SEM (Microgate GmbH, Bolzano, Italy) to measure cognitive decision-making, sustained attention, reactivity, and decision-making in football situations and passes; Polar RS400 (Polar Electro Oy, Kempele, Finland) for measuring heart rate
Slutter et al. [56]	fNIRS (Artinis Brite 24, The Netherlands)	10 Hz	✓			✓		15 penalty kicking task were made in three rounds, each of which consisted of five penalties with the goalkeeper. The pressure increased per round	Preferred	Artificial soccer pitch	Yes - GK	2D kinematics (2 GoPro^®^ cameras − 60 Hz) one for measuring placement and power of the shot. Another GoPro was used to measure the duration for which players looked at the goalkeeper

Common signal processing procedures in EEG studies included re-referencing/down-sampling, passband filtering, artifact suppression (by visual inspection and/or automatically with pre-defined thresholds), rejection of bad channels and rejection of components by independent component analysis (ICA). From the six studies assessing EEG, five [29–31, 53, 54] processed the data in the EEGLAB environment [57]. In this sense, Palucci Vieira et al. [29] used EEGLAB v2020.0 while Li et al. [30] do not specified the version of the toolbox used. The three studies by Piskin et al. [31, 53, 54] used the EEGLAB version 14.1.2b with identical processing methods across studies. As regarding fNIRS studies, processing procedures consisted generally in, as for example, passband filtering, automatic removal of data based on pre-defined thresholds and data transformation. Full details of the procedures for each study can be found in the Tables 3 and 4. Schmaderer et al. [55] used the nirsLab software package v.2019.4, as did Slutter et al. [56] used OxySoft software. Complementary measurements generally consisted in motion kinematics: three studies [31, 53, 54] used wearable IMU systems (myoMOTION, Noraxon), one study used 3D videogrammetry [29] whilst another 2D [56]; both works employed GoPro^®^ cameras for video recording.

**Table 3:** Methodological reporting of EEG and fNIRS acquisition and processing parameters across included studies

Reporting parameter	Collins et al. [52]	Li et al. [30]	Palucci Vieira et al. [29]	Piskin et al. [53]	Piskin et al. [54]	Piskinet al. [31]	Schmadere ret al. [55]	Slutter et al. [56]	*N*° studies reporting (X/*N*)
**Modality**	EEG	EEG	EEG	EEG	EEG	EEG	fNIRS	fNIRS	
***Acquisition setup***									
**Portable/wireless system**	NR	NR	✓	✓	✓	✓	✓	✓	**6/8**
**N° of channels**	6	64	64	65	65	65	21	23	**8/8**
**Electrode positioning system**	10–20	10–20	10/10	10–20	10–20	10–20	10–20 based	NR	**7/8**
***EEG signal processing***									
**Online reference electrode**	Cz	NR	NR	FCz	FCz	FCz	N/A	N/A	**4/6**
**Offline re-referencing**	NR	Avg. mastoids	Common avg.	Common avg.	Common avg.	Common avg.	N/A	N/A	**5/6**
**ICA applied**	No	✓	✓	✓	✓	✓	N/A	N/A	**5/6**
**ICA variant specified**	N/A	Runica Infomax	NR	AMICA	AMICA	AMICA	N/A	N/A	**4/6**
**Auto bad-channel detection**	NR	NR	✓ (kurtosis > 3 SD)	✓ (z-score > 5)	✓ (z-score > 5)	✓ (kurtosis, prob., spec.)	N/A	N/A	**4/6**
**Source- vs. sensor-level analysis**	Sensor	Sensor	Sensor	Source	Source	Source	N/A	N/A	**6/6 (3 S/3So)**
***fNIRS signal processing***									
**Optode layout reported**	N/A	N/A	N/A	N/A	N/A	N/A	8 S/8D; 21 ch	23 ch	**2/2**
**Short-separation channels**	N/A	N/A	N/A	N/A	N/A	N/A	NR	NR	**0/2**
**Motion correction method**	N/A	N/A	N/A	N/A	N/A	N/A	NR (CV only)	TDDR	**1/2**
**Physiological filtering**	N/A	N/A	N/A	N/A	N/A	N/A	BP 0.01–0.2 Hz	BP 0.02–0.5 Hz	**2/2 (BP only)**

**Table 4:** Brain-derived data analysis and associated main outcomes in the included studies

Reference	Brain channels collected	Frequency bands	Signal processing (software and procedures)	Phase(s) of the movement analyzed	Brain-derived outcomes computed	Additional variables	Summary
Collins et al. [52]	T3, T2, C3, C4, P3, P4; Reference: Cz; Ground: spine (Thoracic 2).	alpha: 8–13 Hz	CEAN 400 Acquisition System; Digitized amplified signal at a sampling rate of 256 Hz; Filters from 1 to 100 Hz; Frequency analysis by Fast Fourier Transform (FFT) in a spectrum from 1 to 60 Hz, using a cosine bell window with 256 points; alpha frequency band (8–13 Hz)	Preparatory (-2s)	EEG-derived average power spectral density	Successful and unsuccessful performance of kick	The authors identified that greater activity in the alpha frequency band before a kick can predict successful kicking performance in non-expert individuals
Li et al. [30]	Fz and Cz; Reference: M1 and M2; Ground: AFz	alpha: 8–13 Hz	EEGLAB: re-referenced to the averaged (m1, M2); bandpass (1–30 Hz); extracted epochs (-3000-1000ms); removed bad channels; rejected artifacts above ± 100 µV; ICA; interpolated channels	Preparatory (-2s)	EEG- derived power through Welch estimation method	Learning effect: compare the success rate across the three blocks; Anxiety level: compare between and within subjects during the football penalty task	Success in penalty kicks in a difficult task is characterized by lower power levels of 8 to 13 Hz in the frontal and central regions, indicating effective neuromotor allocation without compromising performance
Palucci Vieira et al. [29]	F3, Fz, F4; C3, Cz, C4; P3, Pz, P4; O1, Oz, O2 Reference: Common average Ground: --	delta: 0.5–3 Hz theta: 4–7 Hz alpha: 8–12 Hz beta: 13–30 Hz gamma: 31–50 Hz	EEGLAB v2020.0: Butterworth band-pass (0.3–50 Hz); notch filter (60 Hz); data re-sampled to 512 Hz; artifacts rejected by (1) visual inspection (2) channels with kurtosis > 3 standard deviations from the mean value, (3) epochs with absolute difference > 150 mV and (4) ICA	Preparatory (-6 to -3 s), approach (-3 to -1 s) and impact phase (-1 s to ball impact)	EEG-derived average power spectral density; ERS/ERD; ERSP and ITC	3D Kinematics: Angular joint (hip, knee and ankle) displacement and velocity; ROM; foot velocity; ball velocity; 2D kinematics: kicking accuracy	Authors claim the association between kicking ball velocity and EEG-derived frontal theta power while kicking accuracy was related to occipital alpha power
Piskin et al. [53]	Clusters of brain sources, whose locations occurred in the parieto-occipital and mid-frontal regions; Reference: FCz; Ground: AFz	theta (4–7 Hz); Alpha (8–10 Hz); Alpha-2 (11–13 Hz); Beta-1 (14–20 Hz)	EEGLAB toolbox (version 14.1.2b): remove sinusoidal line noise; band-pass (3–30 Hz); artifacts rejected by (1) remove robust z-socre > 5; (2) Missing channels were interpolated; (3) data were then re- referenced to a common average and downsampled to 256 Hz; (4) an epoch time window of 3000 ms before and after kick-onset; (5) Baseline correction was performed from − 2500 to − 2000 ms; (6) ICA	Preparatory (-2s); Banckswing and swing: 0–1000 ms; Follow-through: 1000–2000 ms.	ERSP	3D biomechanics of the kick: reliability, movement range and peak acceleration; webcam: Accuracy rate	The authors reveal that the right parieto-occipital cluster demonstrated strong alpha desynchronization after the kick, indicating increased visual demands. The mid-frontal cluster revealed theta synchronization before ball contact and alpha desynchronization beginning in the follow-through phase, indicating executive processing and attentional demands
Piskin et al. [54]	Right posterior and Mid-frontal Clusters (ERSP) AF3, AFz, AF4, F3, F1, Fz, F2 and F4; Pz, P2, P4, P6, P8, POz, PO8, Oz, O2 (MSE); Reference: FCz; Ground: AFz	theta (4–7 Hz); Alpha (8–10 Hz); Alpha-2 (11–13 Hz); Beta-1 (14–20 Hz)	EEGLAB toolbox (version 14.1.2b): remove sinusoidal line noise; band-pass (3–30 Hz); artifacts rejected by (1) remove robust z-score threshold > 5; (2) data were then re- referenced to a common average and downsampled to 256 Hz; (3) the data were epoched from − 3000 to 3000 ms, with baseline correction applied from − 2500 to -500 ms; (4) ICA; (5) All non-brain independent component were subsequently removed from the dataset	From − 3000 to 3000 ms, with baseline correction applied from − 2500 to -500 ms	ERSP and MSE	3D biomechanics (hip flexion, knee flexion, foot external rotation and acceleration) were digitized and processed for complexity analysis in Matlab (MSE)	The authors reveal that injured players exhibit sensorimotor alterations during kicking (differences in posterior alpha and frontal theta oscillations), suggesting compensatory strategies for performing the kicking task, hindering the integration of relevant information
Piskin et al. [31]	Clusters of brain sources, whose locations occurred in the parieto-occipital and frontal regions; Reference: FCz; Ground: AFz	theta (4–7 Hz); Alpha (8–10 Hz); Alpha-2 (11–13 Hz); Beta-1 (14–20 Hz)	EEGLAB toolbox (version 14.1.2b): remove sinusoidal line noise; band-pass (3–30 Hz); artifacts rejected by (1) remove robust z-score threshold > 5; (2) data were then re- referenced to a common average and downsampled to 256 Hz; (3) the data was epoched to -0-2500 ms from pass onset to focus on post-onset; (4) ICA	Post-onset (0–2500 ms) (ERSP)	ERSP and MSE	3D biomechanics (hip flexion, knee flexion, foot external rotation and acceleration) were digitized and processed for complexity analysis in Matlab (MSE)	The authors revealed that more experienced players exhibit greater passing accuracy, which may indicate the influence of visuospatial and attentional strategies evidenced by alpha parieto-occipital desynchronization and theta frontal synchronization
Schmaderer et al. [55]	Frontomedial: channel (ch) 11, 17–19; ventrolateral: ch 15–16, 20–21; dorsolateral: ch 1–10, 12–14.		nirsLab (2019.4): band pass filter (low cut-off frequency: 0.01 Hz; high cut-off frequency: 0.2 Hz); rejection of channels with variance > 7.5%; The first 45s were considered and divided into three time blooks 15s each; transformation to hemodynamic data	Baseline (15s); After the start of the test (15 to 30s)	Average oxyhaemoglobin	Reactivity and decision-making to visual and auditory stimuli; cognitive decision-making, sustained attention, reactivity, and decision-making in football situations, passes, and heart rate	The authors revealed increased activity in the prefrontal region during novel stimuli compared to sport-specific tasks, a result of the automaticity acquired by experts in the field
Slutter et al. [56]	Motor cortex: channel (ch): 1–4; right prefrontal cortex: ch 9–12; left prefrontal cortex: ch 9–12; left temporal cortex: ch 13–16; right and left dorsolateral prefrontal cortex: ch 17–18, respectively.		OxySoft - transform fNIRS signals; Scikit-learn package; Butterworth bandpass (0.02–0.5 Hz); Temporal derivative distribution repair method for artifact rejection; Tukey’s biweight function; data were then re- referenced to baseline; rejection of channels with ultra-low FNIRS activity; the last 15 s of the resting period was used to subtract from datapoints; removal of data based on correlation coefficient (> 0.4) between O2Hb and HHb signals per trial-channel	Prepatory (-5s) before the researcher’s signal	O2Hb concentration; average O2Hb activation in each region; Averaged prefrontal cortex activation (left and right); prefrontal cortex asymmetry; connectivity index	Small questionnaire: Sport Competition Anxiety Test; Sport Anxiety Scale; Successful and unsuccessful performance of kick; placement and shot power; goalkeeper-looking duration	The study revealed distinct brain activation between anxious players (greater activation and asymmetry in the prefrontal cortex and less activation in the motor cortex) and non-anxious players, reinforcing the logic of the neural efficiency theory, which suggests greater activity in relevant areas and suppression of irrelevant ones

Four of the six EEG studies used wireless or portable equipment [29–31, 53, 54], one had a fixed, wired amplifier [52], and one did not clearly specified whether the system was portable [30]. The number of electrodes in the EEG setups ranged from as few as six selected electrodes [52] to highly detailed designs of 64 or 65 electrodes [29, 31, 53, 54]. All but one of the studies [52] performed their setpoint adjustments after recording, and five of the six [29–31, 53, 54] used a common-average method for this purpose. Five of the six studies [29–31, 53, 54] used ICA to clean the data; of these, three [31, 53, 54] used AMICA and one [30] used Runica Infomax. Four of the six studies automatically identified and removed low-quality EEG channels. None of the studies used artifact subspace reconstruction algorithm. Three of the six studies [31, 53, 54] analyzed the EEG signals at their brain origin using ICA and dipolar modeling, while the other three [29, 30, 52] worked with the signal as captured by the sensors. In the fNIRS studies, none used short-range channels to account for bodily processes such as pulse or respiration. One of the two studies [56] used motion correction with the TDDR method, along with a method to verify data quality based on the matching of different parts of the signal, allowing approximately 41% of the data to be retained for each channel and each attempt. Filtering for physiological noise was limited to reducing frequencies between 0.01 and 0.2 Hz [55] or between 0.02 and 0.5 Hz [56], and none of the studies applied additional filtering to compensate for the effects of heart rate or respiration. Table 3 provides a detailed overview for all the studies.

### Qualitative synthesis of evidence

Table 4 presents the key outcomes derived from the included papers. Firstly, as regarding the EEG studies evaluating ball kicking [29–31, 52–54], four main dependent variables were computed across such investigations. These consisted of (i) the event-related spectral perturbation (ERSP), being the most frequently reported outcome (4 of 6 studies; [29, 31, 53, 54]), followed by (ii) EEG power (3 studies; [29, 30, 52]), (iii) multiscale entropy (MSE; 2 studies; [31, 54]), and less frequently (iv) the inter-trial coherence (ITC; 1 study [29]).

The early study by Collins et al. [52] reported that in non-expert participants (i.e., non-soccer players), successful attempts (i.e., ball travelled through a 30-centimeter-wide channel) resulted in higher pre-kick alpha power at temporal sites than unsuccessful attempts. Palucci Vieira et al. [29] reported that the kicking velocity was associated with frontal theta power during the impact phase, while kicking accuracy was associated with occipital alpha power during the preparatory phase. In a similar study, Li et al. [30] reported that lower 8–13 Hz power at the frontal and central regions was associated with successful penalty kicks.

Piskin et al. [53] initially investigated the measurement error of the EEG to assess ball kicking and reported that the right parieto-occipital cluster demonstrated strong alpha desynchronization after kick, while the mid-frontal cluster revealed theta synchronization before ball contact and alpha desynchronization beginning in the follow-through phase. These variables were shown to have moderate to excellent reliability. Another study by Piskin et al. [31] reported that, compared to novice soccer players, expert soccer players showed greater passing accuracy and exhibited earlier and stronger alpha desynchronization at the right parieto-occipital region prior to ball contact, as well as stronger frontal theta synchronization at ball contact. In a third separate experiment evaluating the role of injuries on kicking-derived EEG signals, Piskin et al. [54] reported differences in cortical activation between participants with injuries and those without injuries; healthy participants exhibited stronger alpha desynchronization post-kick, whereas injured participants showed stronger theta synchronization at the mid-frontal cluster during both the onset of kick and the post-onset period. These findings suggest that injured athletes maintained accuracy by using compensatory strategies for the kicking task, which hindered the integration of relevant information.

**Fig. 2:**
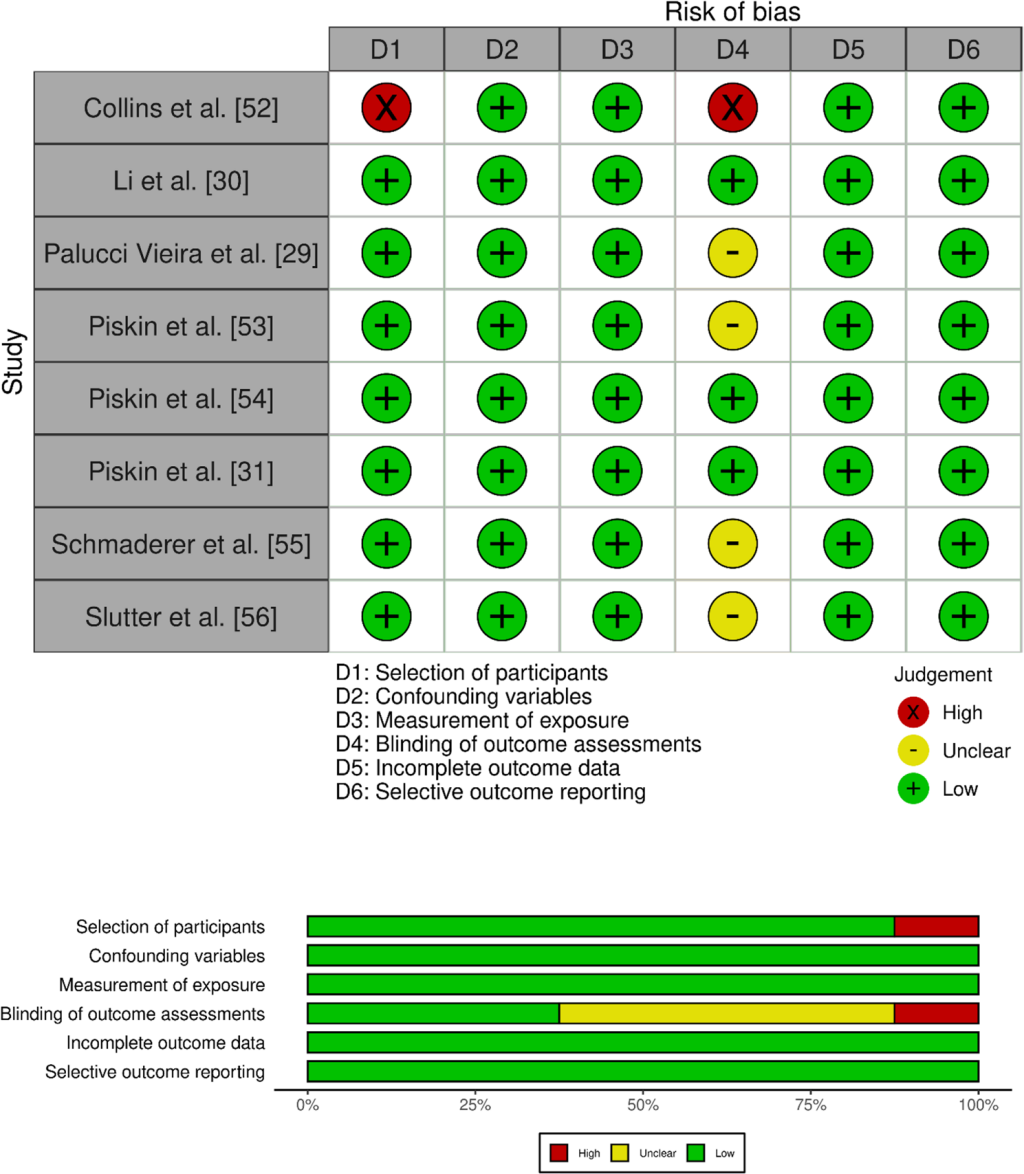
Outcomes of the risk-of-bias analysis assessed using the RoBANS tool, separate for each included study (above) as well as across all studies pooled (below)

Finally, regarding the evidence obtained in the studies [55, 56] using specifically the fNIRS technology to evaluate the ball kicking movement, Schmaderer et al. [55] reported that compared to known stimuli (i.e., sport-specific test), unknown or novel stimuli showed significantly higher prefrontal activity while Slutter et al. [56] reported differences in brain activation between anxious and non-anxious players, with anxious players exhibiting greater activation and asymmetry in the prefrontal cortex and lower activation in the motor cortex. The outcomes of the reporting completeness are presented in Table 5 [Additional file 2] and the outcomes of the risk-of-bias analysis are presented in Fig. 2. The reporting completeness assessed using the STROBE checklist was reported to be high for all the included studies. Conversely, the risk-of-bias analysis using the RoBANS tool revealed that the item blinding of outcome assessments showed the highest uncertainty across studies (i.e., 4/8 studies with unclear risk [29, 53, 55, 56]) while all other items showed a low risk of bias in the majority (> 75%) of reports.

### Gap map

**Fig. 3:**
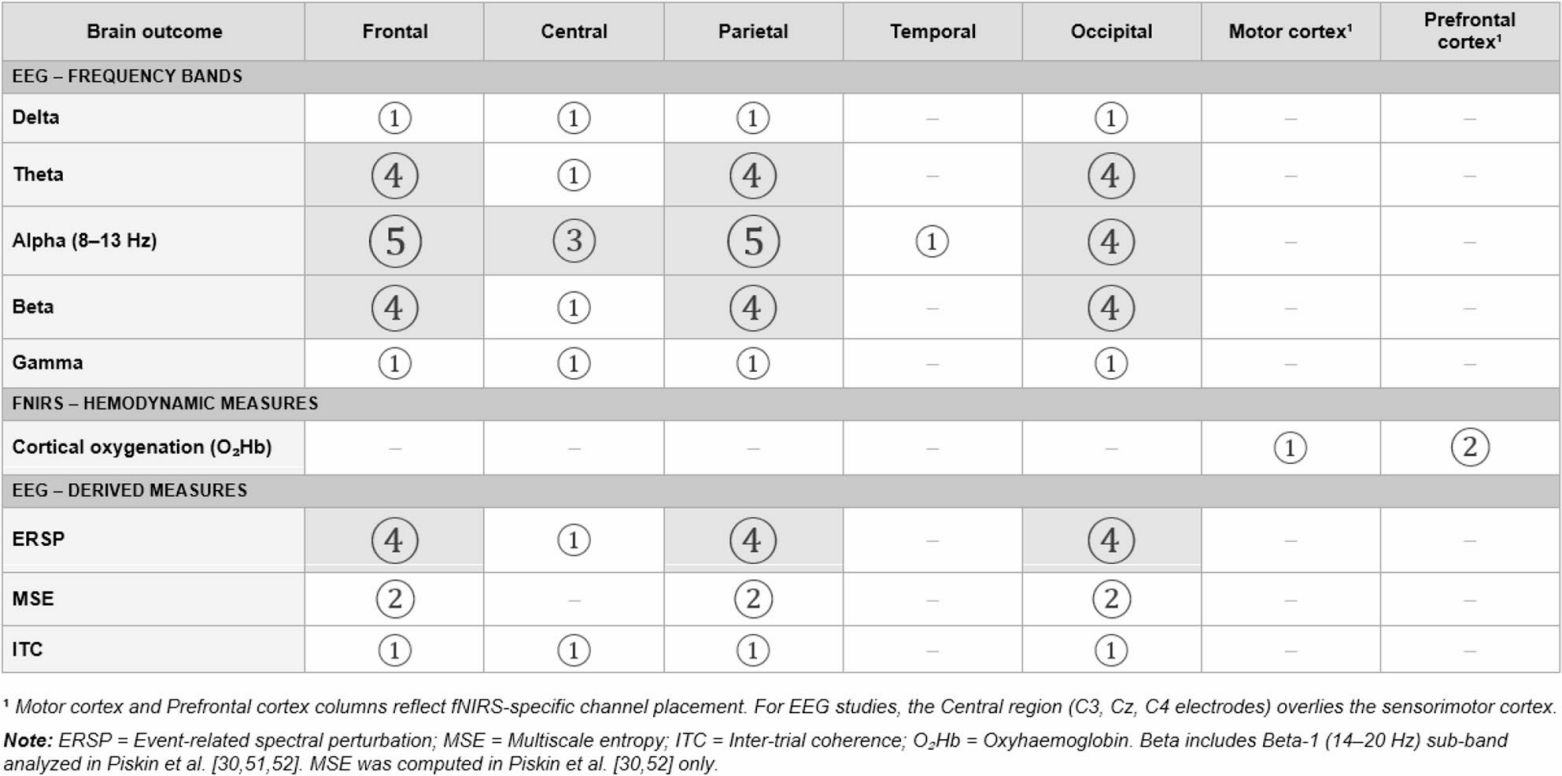
Interaction between the brain-derived outcomes investigated and the brain regions explored in the included studies. Open circles represent the number of studies. Shaded cells indicate evidence from ≥ 3 studies. – = No evidence available

**Fig. 4:**
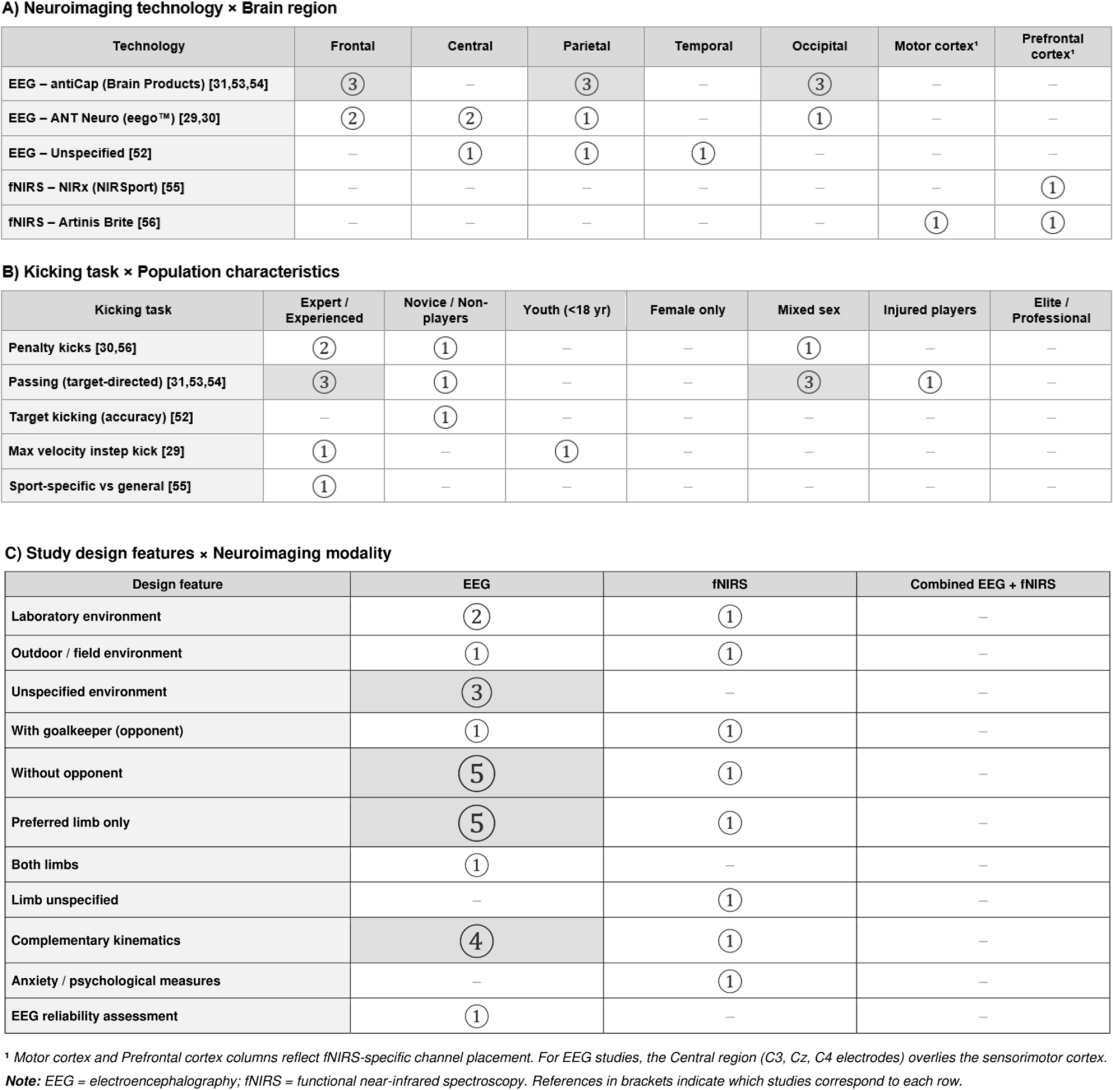
Evidence gap map regarding the neuroimaging technologies/modalities, brain regions, and key study design features across the included studies. Open circles represent the number of studies found. Shaded cells indicate evidence from ≥ 3 studies. – = No evidence available

Figure 3 illustrates the interaction between the brain parameters studied (frequency bands and cortical activation measures) and the brain regions explored in the included studies. In addition, Fig. 4 presents the gap mapping according to neuroimaging technologies, brain regions of interest, and key design features of the included studies.

The alpha band oscillation was the most researched neural marker (Fig. 4A), including data in the frontal (*n* = 5 studies; [29–31, 53, 54]), parietal (*n* = 5 studies; [29, 31, 52–54]), occipital (*n* = 4 studies; [29, 31, 53, 54]), and central (*n* = 3 studies; [29, 30, 52]) brain regions. Limited data were found on alpha band activity in the temporal region (one study; [52]). Theta band oscillations were examined in the frontal, parietal and occipital (*n* = 4 studies for all the these three regions; [29, 31, 53, 54]), while available data on theta band were limited in the central region (one study; [29]). The beta frequency band (including the beta-1 sub-band, 14–20 Hz) was also investigated in the frontal, parietal, and occipital regions (*n* = 4 studies each; [29, 31, 53, 54]), and with limited evidence in the central region (one study; [29]). For delta and gamma frequency bands, only limited data were found, each examined in a single study [29] in the frontal, central, parietal, and occipital regions. Cortical oxygenation (fNIRS) was studied in the frontal cortex (two studies; [55, 56]) and to a limited extent in the motor cortex (one study; [56]), with no fNIRS data available for the parietal or occipital regions.

Regarding population characteristics and study methodology, results for experienced players during ball passing drills were the most researched (3 studies; [31, 53, 54]). Some data were also available for penalty kicks with experienced players (2 studies; [30, 55]), while limited data existed on target kicking with novice participants (1 study; [52]), and maximum velocity instep kicks with young players (1 study; [29]). No data were found (Fig. 4B) for exclusively female samples, professional/high-level or elite participants, samples of children or pre-adolescents, or for game-play conditions during brain signal acquisition. The majority of studies (6 out of 8) did not include any opponents [30, 31, 52–55], and only two studies included goalkeepers as opponents [29, 56]. On-field testing conditions were also scarce (2 studies; [29, 56]) while more studies were available using laboratory environments. Only limited evidence on the use of non-preferred limb was identified (1 study; [52]).

As concerning the data acquisition technology, six studies employed EEG [29–31, 52–54], while two used fNIRS [55, 56] as their primary neuroimaging modality. No study combined both technologies simultaneously. Additional technologies were used to measure kinematics in five studies [29, 31, 53, 54, 56], while psychological measures (e.g., anxiety) were assessed in only one study [56]. Finally, only one study [53] formally evaluated the reliability of EEG signal measurements during the ball kicking task (Fig. 4C), as no equivalent reliability data are available for fNIRS-based protocols.

## Discussion

This systematic review synthesized evidence on brain-derived signals measured during soccer ball-kicking tasks and related them to performance outcomes and contextual constraints. Most of the evidence found here refers to brain-performance associations. Convergent findings indicate that successful and/or higher-quality kicking performance is generally accompanied by phase-specific cortical dynamics, particularly within frontal (attentional/motor-programming), sensorimotor/central (motor control), and parieto-occipital (visuospatial) regions. In EEG studies, performance was most consistently linked to modulations in theta and alpha bands across distinct phases of the kick (preparation, approach/execution, follow-through), whereas fNIRS studies emphasized the role of prefrontal and motor-cortex oxygenation patterns under pressure/anxiety or varying cognitive demands. However, blinding aspects were often poorly accounted for. Using the gap map method, it was possible to observe that currently there are no literature studies using EEG–fNIRS as part of the experimental paradigm as well as only one-time studies (i.e., limited evidence) were identified evaluating, as for example (i) the neural mechanisms underlying leg dominance and interlimb asymmetry during kicking; (ii) measurement error (e.g., reliability) of brain-derived metrics and (iii) kicks outside penalty area. In the following paragraphs we will offer interpretations to the main findings of the present systematic review as well as possible directives for future investigations.

### Neurophysiological correlates of kicking success and performance outcomes

Among included studies, there was a great interest in the topic of neurophysiological correlates of kicking success and performance outcomes. In penalty-kick contexts, successful trials were associated with lower 8–13 Hz power in frontal and central regions during motor preparation (approximately the final 2 s pre-kick), suggesting a more efficient pre-action state and reduced costly cortical engagement for planning/control when execution is successful [30]. These results align with efficiency-oriented interpretations in which skilled performance is supported by selective recruitment of task-relevant networks without excessive prefrontal/sensorimotor activation that might reflect conscious control or maladaptive attentional capture [30].

Evidence also links brain signals to continuous performance dimensions (velocity and accuracy), rather than binary success alone. In youth sub-elite players performing instep shots from longer distance with a goalkeeper present, frontal theta activity was associated with ball velocity, whereas occipital alpha activity during preparation was associated with accuracy (mean radial error) [29]. These associations map well onto a dual-demand structure of soccer kicking. Generating ball speed requires coordinated high-force multi-joint sequencing that may depend on frontal/cognitive control signals at critical instants, while accuracy depends heavily on visuospatial processing and stabilization of perceptual information (indexed here by posterior alpha modulation). Importantly, early evidence from a foundational study using a simpler task suggested that, in non-soccer participants, greater pre-kick alpha at temporal sites also predicted successful execution [52]. While that early result should be interpreted cautiously due to task simplicity, participant characteristics, alongside its high risk of bias in some items, it suggests that successful kicking can be preceded by measurable oscillatory differences even in relatively novice performers, albeit with topographies and interpretations that may differ from later mobile-neuroimaging paradigms.

Of note, one area underexplored in the studies evaluating the role of brain signals in the kinematics and outcomes of ball kicking refers to the asymmetries commonly observed. While there is extensive evidence as concerning the presence of asymmetry in the kinematics and outcomes of ball kicking, favoring the dominant side across investigations including various ages, genders and playing levels [58–63], the central mechanisms likely involved remain unclear. This is confirmed here since among the studies included in the present systematic review, only one considered kicks with the dominant and non-dominant limbs (Table 2); in this isolated study authors provided a brief mention that there was no main effect for preferred vs. non-preferred kicking limb on brain-derived signals computed as well as it was not one of the main objectives of the such study to evaluate asymmetries [52], thus implying existence of only limited evidence regarding this issue.

### Visuospatial and attentional strategies indexed by posterior alpha and frontal theta dynamics

In a reliability-focused EEG study using source-derived approaches, consistent parieto-occipital (alpha desynchronization) and mid-frontal (theta synchronization) dynamics were observed across sessions, supporting their candidacy as stable measure of directed pass-kicks [53]. Extending this, expertise comparisons showed that experienced players demonstrated higher pass accuracy alongside earlier/stronger parieto-occipital alpha desynchronization prior to ball contact and stronger frontal theta synchronization around ball contact [31]. These findings are consistent with an expertise-related refinement of visuospatial attention (posterior alpha) and task-focused control/monitoring (frontal theta), potentially reflecting more efficient allocation of resources to extract target information and stabilize the sensorimotor plan.

Complementary evidence from fNIRS further supports the broader principle that familiarity/automaticity reduces prefrontal load. In a sample of semi-professional players, sport-specific (familiar) cognitive tasks elicited lower prefrontal activity changes than general (more novel) cognitive tasks, consistent with the interpretation that learned automatisms reduce reliance on effortful prefrontal processing [55]. Although this study assessed perceptual-cognitive tasks rather than solely biomechanics-focused kicking outcomes, it provides converging support suggesting that when task demands are familiar and well-trained, the system may achieve performance with lower prefrontal control signatures.

### Pressure, anxiety, and injury as perturbations of neural efficiency during kicking

In an ecologically oriented penalty study using fNIRS, anxiety was associated with higher prefrontal activation and altered lateral asymmetry, alongside lower activation in the motor cortex; these patterns were linked to missed penalties and to anxious states, consistent with a choking-under-pressure interpretation emphasizing task-irrelevant prefrontal engagement [56]. Injury status (ACL reconstruction) similarly appears to be associated with altered cortical dynamics and movement variability during target-directed kicking. In the included injury comparison study, injured players exhibited distinct posterior alpha and frontal theta oscillatory patterns relative to healthy players, interpreted as compensatory attentional strategies that may support maintained accuracy at the expense of efficient visuospatial integration [54]. Possibly, soccer kicking can be understood not only as a biomechanical skill but also as a neurophysiological behavior that may be reorganized after injury, potentially contributing to persistent performance deficits or altered coordination strategies even when athletes have returned to play [54].

### Limitations and future research

Currently, it is not uncommon to observe elite soccer players taking penalty kicks with a small jump just before contacting the ball [64], potentially aiming to delay their final movement and possibly make an online adjustment to the direction of the kick based on the goalkeeper’s movements. This could in some ways contradict part of the literature that indicates that information about which side the goalkeeper decides to dive should be obtained by the penalty taker early in the approach run [65]. Among the studies included here, only one indicated that participating players could adopt an approach run with varying speeds (i.e. slow down) if they wanted to [56]. However, to date there have been no studies that have analyzed brain signals specifically in relation to this contemporary type of kicking strategy using small jump/hop immediately before ball contact in shooting.

Another important limitation identified in the present review study is that a goalkeeper attempting to block shots was used in only two of the included studies (see Table 2). This might reduce the usefulness of the evidence collated here to “real-world” conditions. In fact, previous experimental studies indicate that unopposed testing scenarios have only limited value for predicting actual game performance in terms of ball kicking ability [66–68]. On the other hand, the (unknown) trade-off between ecological validity and motion artifacts cannot be also overlooked. Arguably, it is not uncommon to find reports indicating that soccer kicking represents an asymmetrical task (involving a stabilizing leg and a kicking leg), and the lack of neurophysiological data on inter-hemispheric differences is an important gap across studies. As such, future research is advisable to investigate the neural mechanisms underlying leg dominance and interlimb asymmetry during kicking. Notwithstanding, the role of vision has been also widely documented for having a direct impact on the outcomes of kicks in football [69–71]. Meanwhile, according to the present systematic review only half of the literature studies considered brain-derived measurements collected from the occipital region.

The principal limitation of the current evidence base is its relatively small size (eight original research articles published until the date of the searches) and high heterogeneity across tasks, contexts, technologies, and analytic methods, which precluded quantitative synthesis and limits the confidence of mechanistic claims. Although it is noteworthy (i.e., not limited) the amount of research on the specific topic of assessing the brain-kicking performance associations [29–31, 52, 56], there is also substantial diversity in tasks (short pass-kicks vs. long instep shots vs. penalties), environments (laboratory vs. artificial pitch vs. natural grass), and opponent presence, all of which likely modulate cortical demand profiles and would indeed complicate cross-study synthesis. One potential explanation for the small number of studies until the time of writing of this review is that the proposed experiments were probably only made possible by the most recent technological advances in the area (i.e. 88% of studies published after 2020 – see Table 1); in fact, until shortly before that (in 2018), it was reported in a position manuscript by Perrey and Besson [17] an extreme difficulty in dealing with signal quality issues using both methodologies (EEG and fNIRS) in sports contexts. Future research should use more standardized task taxonomies with explicit manipulation of ecological constraints, preregister primary neurophysiological hypotheses to reduce analytic flexibility, expand beyond sub-elite samples and include women and youth players, and converge on harmonized reporting for mobile EEG/fNIRS in sport movements, including artifact quantification and sensitivity analyses. Since visual inspection is common in processing brain-derived data, blinding of outcome assessments (e.g., as concerning key study personnel) should be also more carefully treated in future studies.

Recently, a comparative study analyzing consumer versus research-grade EEG systems during arbitrary real-world tasks demonstrated that consumer EEG systems exhibit greater noise-related artifacts in real-time data measurement - particularly in the anterofrontal brain regions where large amounts of neurometric measurements were obtained - lower data consistency and greater computational instability [72]. These findings underscore the importance of transparent reporting of system specifications in selecting data collection systems that meet the analytical requirements and environmental constraints of the study. Regarding future validated options for mobile/portable EEG frameworks, the use of multi-stage pipelines could offer promising results (e.g., ARS [73, 74], o/CFo-CLEAN [75, 76] and VMD [77] algorithms). Given the rapid body movements involved in kicking a soccer ball, conventional assumptions in ICA may require validation, and future studies should compare their artifact correction methods with these newly developed EEG pipelines. Additionally, the lack of short-spacing channels in fNIRS studies is increasingly recognized as a critical deficiency, as these channels are essential for monitoring systemic hemodynamic variables during whole-body motor tasks. Furthermore, the choice between source-level and sensor-level analysis must be clearly justified before starting the analysis.

Finally, the decision to exclude studies of the present review conducted without reporting of any ethical aspects was based on a recent trend in systematic reviews [41, 51, 78–80], as recommended previously elsewhere in Vergnes et al. [44]. This exclusion criterion was also adopted in order to avoid any possible conflict with the institutional and national guidelines on research ethics as concerning the institution hosting the project. On the other hand, we confirm that none among the reports assessed for eligibility were excluded specifically on the basis of this criterion (see Fig. 1). As such, the potential issues it may have introduced in the current systematic review synthesis (e.g. bias the evidence base toward certain journals/regions rather than study quality) is likely negligible.

### Practical applications

The emerging evidence suggests that training and rehabilitation programs may benefit from the cognitive–sensorimotor processes indexed by frontal theta and posterior alpha dynamics. For performance training, coaches and practitioners could integrate practice designs that promote stable visuospatial attention and automated execution under accuracy constraints, while systematically introducing pressure elements to reduce maladaptive prefrontal over-engagement associated with anxiety and missed penalties [56]. For injury rehabilitation, monitoring of task-related cortical dynamics and movement variability during standardized pass-kick tasks may help identify compensatory attentional strategies and guide progression toward more efficient visuospatial–sensorimotor integration before full return to competition [54].

## Conclusions

Evidence from a small but growing literature indicates that soccer ball-kicking performance is associated with measurable, phase-specific cortical dynamics, most consistently involving frontal theta and parieto-occipital alpha modulations during preparation and execution. Across studies, better performance and expertise tend to align with patterns consistent with efficient attentional allocation and visuospatial processing, whereas anxiety and injury contexts appear to shift cortical engagement toward potentially compensatory, less efficient control strategies. While these findings are interesting, they remain preliminary due to the quantity of studies, modest sample sizes, and heterogeneous methods. Given the complementary nature of temporal (EEG) and spatial (fNIRS) resolutions, the current systematic review with gap map identified that future research should attempt to evaluate ball kicking using both technologies concomitantly, as none of the studies included here has done this type of analysis until the moment.

## Supplementary Information

Supplementary Material 1. PRISMA 2020 checklist completed for the present systematic review on brain-derived signals related to ball kicking movement in soccer and technologies employed.

Supplementary Material 2. Reporting completeness outcomes using STROBE checklist.

## Data Availability

Similarity and AI writing reports for the present paper can be found for download in [https://doi.org/10.5281/zenodo.18716878](https:/doi.org/10.5281/zenodo.18716878) . All raw data supporting this systematic review are derived from previously published studies, which have been cited in the text and reference list. Additional processed data that support the findings of the current review are available from the corresponding author on reasonable request.
