# Supplementary material for: Brain-derived signals related to ball kicking movement in soccer and technologies employed: a systematic literature review with gap map
Source: BMC Sports Sci Med Rehabil. 2026 Apr 1;18:234. doi: 10.1186/s13102-026-01676-y (PMC13169947; doi:10.1186/s13102-026-01676-y)
Supplement: Supplementary file 2 — Supplementary Material 2. Reporting completeness outcomes using STROBE checklist. [file 13102_2026_1676_MOESM2_ESM.pdf]

Table 5. Outcomes of the reporting completeness assessed using the STROBE checklist separate for each included study.

Study	Q1	Q2	Q3	Q4	Q5	Q6	Q7	Q8	Q9	Q10	Overall (∑)
Collins et al. [52]	1	1	1	1	1	0	1	1	1	0	8
Li et al. [30]	1	1	1	1	1	1	1	1	1	1	10
Palucci Vieira et al. [29]	1	1	1	1	1	1	1	1	1	1	10
Piskin et al. [53]	1	1	1	1	1	1	1	1	1	1	10
Piskin et al. [54]	1	1	1	1	1	1	1	1	1	1	10
Piskin et al. [31]	1	1	1	1	1	1	1	1	1	1	10
Schmaderer et al. [55]	1	1	1	1	1	1	1	1	1	1	10
Slutter et al. [56]	1	1	1	1	1	1	1	1	1	1	10

Note: 1 = completed; 0 = incomplete. The items Q1 to Q10 corresponds respectively to the items 1, 3, 6, 8, 12, 14, 18, 19, 20 and 22 derived from STROBE checklist.
